# Comparative Metabolomics of Reproductive Organs in the Genus *Aesculus* (Sapindaceae) Reveals That Immature Fruits Are a Key Organ of Procyanidin Accumulation and Bioactivity

**DOI:** 10.3390/plants10122695

**Published:** 2021-12-08

**Authors:** Alison Green, Guillermo Federico Padilla-Gonzalez, Methee Phumthum, Monique S. J. Simmonds, Nicholas J. Sadgrove

**Affiliations:** 1 Royal Botanic Gardens, Kew, Richmond Surrey, London TW9 3AD, UK; avfgreen@gmail.com (A.G.); f.padilla@kew.org (G.F.P.-G.); methee.phu@mahidol.ac.th (M.P.); m.simmonds@kew.org (M.S.J.S.); 2 Department of Pharmaceutical Botany, Faculty of Pharmacy, Mahidol University, 999 Phutthamonthon Sai 4 Rd, Salaya, Phutthamonthon District, Nakhon Pathom 73170, Thailand

**Keywords:** *Aesculus*, liquid chromatography, mass spectrometry, metabolomics, bioactivity

## Abstract

Fruit from *A. hippocastanum* L. are used commercially for chronic venous insufficiency (CVI). The isomeric mixture of pentacyclic triterpenoid saponins (β-aescin) exert anti-inflammatory effects. Hence, research has focused on β-aescin, yet the diversity, accumulation, and bioactivity of organ-specific secondary metabolites represent missed pharmacological opportunities. To this end, we applied an untargeted metabolomics approach by liquid chromatography—tandem mass spectrometry (LC–MS/MS) to the chemical profiles of flowers, immature fruits, and pedicels from 40 specimens across 18 species of *Aesculus*. Principal component analysis (PCA), orthogonal partial least squares (OPLS-DA), and molecular networking revealed stronger chemical differences between plant organs, than between species. Flowers are rich in glycosylated flavonoids, pedicels in organic acids and flavonoid aglycones, and immature fruits in monomeric flavan-3-ols and procyanidins. Although a high diversity of flavonoids and procyanidins was observed, the relative amounts differed by plant organ. Fruit extracts demonstrated the strongest antifungal (*Saccharomyces cerevisiae*) and antioxidant activity, likely from the procyanidins. Overall, secondary metabolite profiles are organ-specific, and fruits accumulate antifungal and antioxidant compounds. Due to the chemical similarity between species, similar effects may be achieved between species. This creates incentives for further exploration of the entire genus, in bioprospecting for potential therapeutic leads.

## 1. Introduction

The genus *Aesculus* (Sapindaceae, formerly Hippocastanaceae), more commonly referred to as horse chestnut or buckeye tree, consists of around 15–20 species and numerous subspecies or hybrids [1,2]. Famed for their broad leaves, distinctive spring flowers, sticky winter buds and autumn fruits known as conkers, the genus is one of the Northern Hemisphere’s most popular ornamental trees. Aside from the aesthetic beauty of the trees, they are also of benefit to the environment, and their extracts confer therapeutic effects in people [3]. Due to their hardy properties, taxa from *Aesculus* can be grown in a wide range of environments such as polluted areas where they act as bio-monitors of soil quality and remediate soils by taking up heavy metals [3,4]. Furthermore, in traditional and modern natural pharmacopoeias the fruits of *Aesculus* are extracted into boiling water and used to treat vascular conditions, topically and by ingestion. Other less known applications include improvement of obesity, gastrointestinal problems, skin disorders, rectal afflictions, rheumatism, and headaches [3]. The purified seed extract is still used today to treat chronic venous insufficiency (CVI) where a lack of venous tone causes blood to back flow and create swelling, pain, and oedema [5,6,7].

Underpinning these medical uses are a diverse range of phytoconstituents with known pharmacological properties [3]. Extracts from the fruits of *Aesculus* are rich in coumarins, fraxin, procyanidins, kaempferol, and quercetin derivatives [8,9,10,11]. However, the ingredient that is commonly cited as important in therapeutic outcomes is a mixture of triterpene saponins called aescin (syn. escin), which is extracted from the mature seeds of *A. hippocastanum* L. [6,12]. Commercially, the aescin molecule is modified to allow uptake from the gastrointestinal track [7]. Within the aescin mixture, the fraction of C22-*O*-acetyl saponins, which are known as β-aescin, is considered more important in therapeutic effects. This latter fraction increases the secretion of anti-inflammatory mediators at the site of injury and decreases pro-inflammatory mediators such as adenosine triphosphate (ATP) [6]. Decreasing intra-cellular ATP leads to increased prostaglandins, particularly F2, and platelet activating factor, allowing vascular repair [5]. On top of this, β-aescin targets calcium ion channels, which leads to increases of calcium uptake in muscle tissue. This rise in intracellular calcium augments the contractile force of the muscle, leading to an increase in venous tone and improved blow flow [5,13]. Overall, this lowers the risk of complications from CVI and improves vascular health.

Aside from aescin other chemical constituents within horse chestnuts have bioactivity [14,15,16,17], i.e., the similarly named aesculin, not to be confused with aescin, is a coumarin glycoside that is well known for anticancer and antimicrobial effects [18,19]; fraxins have hepatoprotective properties and the ability to positively affect the immune system [18,20]; procyanidins have long been associated with health benefits as antioxidants, vascular protective compounds [21], and are protective against neurodegenerative diseases [22]; and kaempferol and quercetin derivatives inhibit biofilm formation and alleviate pain and inflammation [23]. Overall, the combination of the bioactive compounds gives horse chestnut extracts numerous beneficial effects.

However, despite the diverse chemistry and wide range of conditions that extracts from *Aesculus* in theory provide benefits to, research and clinical studies are mainly limited to aescin [12]. Therefore, the diversity, accumulation, and bioactivity of organ- and species–specific secondary metabolites has been neglected, creating missed pharmacological opportunities. To this end the following study aimed to perform a comparative metabolomics study of reproductive organs (i.e., flowers, pedicels, and immature fruits) of 40 taxa from 18 species of the genus *Aesculus* and examine their antioxidant and antifungal potential in-vitro.

## 2. Results

### 2.1. Metabolic Profiling of Reproductive Organs

**Figure 1:**
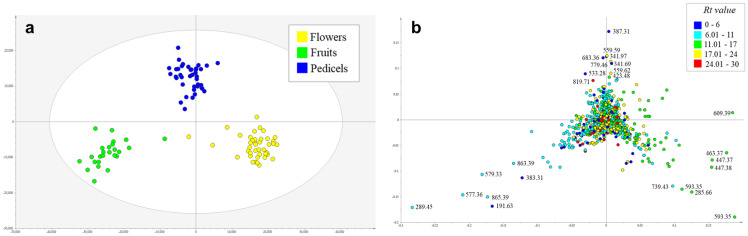
OPLS-DA scores plot (**a**) and loadings plot (**b**) based on metabolic profiling of 106 plant extracts obtained from flowers, immature fruits, and pedicels of 40 taxa from 18 species of *Aesculus*. Discriminant metabolites of each organ are labelled with their *m/z* value in the loadings plot.

Metabolic profiling by UHPLC-UV–MS/MS of crude ethyl acetate extracts from 154 samples obtained from reproductive organs of the genus *Aesculus* (flowers: 41 samples, fruits: 25 samples, pedicels: 40 samples and quality control: 48 samples) detected 1131 mass features in the negative ionization mode. PCA, including all mass features detected in the negative mode (Figure S1), showed a weak clustering tendency by plant organ, suggesting a differential accumulation of metabolites by plant part. To explore those differences, a supervised analysis by OPLS-DA (R^2^Y = 0.917 and Q^2^ = 0.779) was performed using the plant organ as Y variable. This analysis revealed a clear segregation among flowers (Figure S2), immature fruits and pedicels based on their chemical composition (Figure 1a). Analysis of the OPLS-DA loadings plot (Figure 1b) and manual inspection of LC–MS/MS data identified the discriminant metabolites preferentially accumulated in each organ. Interestingly, compounds from different chemical classes were accumulated in each organ (Table 1). The flower extracts showed a higher accumulation of glycosylated flavonoids, such as rutin and kaempferol *O*-rutinoside (Table 1, Figure 1b). On the other hand, the immature fruits were rich in epicatechin and other procyanidins, while early eluting compounds (Rt values = 0–6 min), representing free glycosides and organic acids, were accumulated in higher proportion in the pedicels when compared to the other organs (Figure 1b, Table 1).

**Figure 2:**
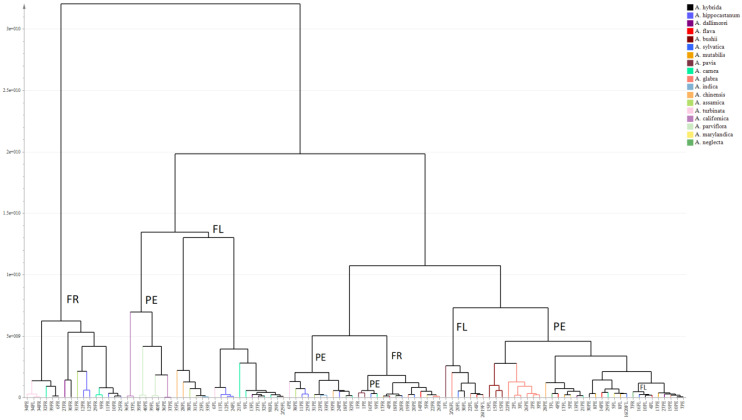
HCA based on metabolic profiling of 106 plant extracts obtained from flowers, immature fruits, and pedicels of 40 taxa from 18 species of *Aesculus* showing a clustering tendency more related to the plant organ than to the species identity. FR: immature fruits, PE: pedicels and FL: flowers.

In contrast, a high degree of inconsistency was seen across different species of *Aesculus* and as such no key discriminating metabolites were found (Figure 2). The hierarchical clustering analysis (HCA) in Figure 2 shows that little differences can be seen across species with the higher chemical differences being attributed to the plant organ than to the species identity.

**Figure 3:**
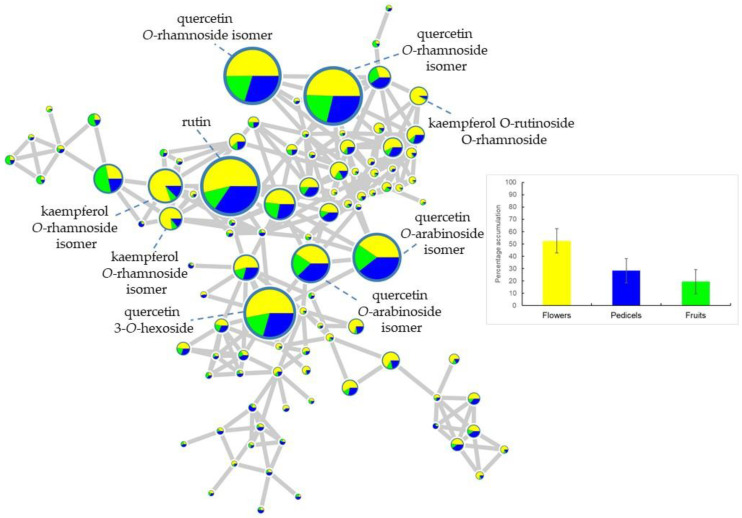
Molecular network showing the diversity of glycosylated flavonoids and accumulation patterns in the reproductive organs of 40 taxa from 18 species of *Aesculus* analyzed by LC–MS. Nodes represent the metabolites detected in the plant extracts with pie charts indicating the accumulation of each metabolite in the three organs (yellow shading for flowers, blue for pedicels, and green for immature fruits). The size of the node denotes the ion intensity detected in the negative ionization mode. Bar chart summarizes the percentage accumulation value of all nodes associated to the same “molecular family”.

**Figure 4:**
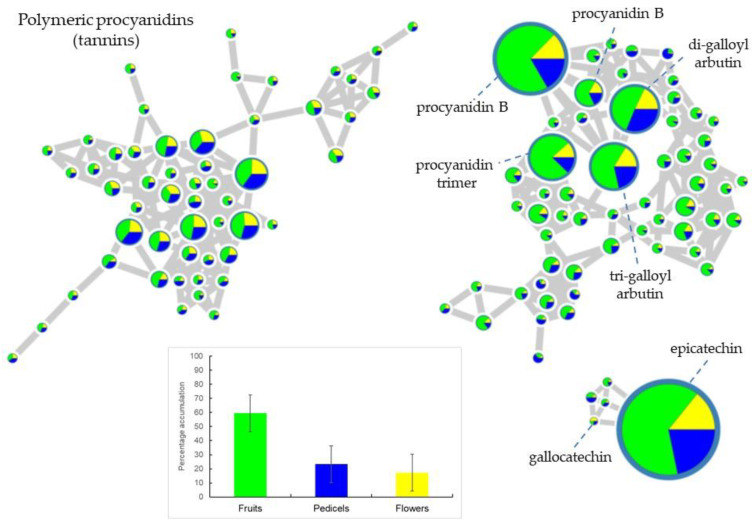
Molecular network showing the diversity of procyanidins and associated metabolites and their accumulation patterns in the reproductive organs of 40 taxa from 18 species of *Aesculus* analyzed by LC–MS. Nodes represent the metabolites detected in the plant extracts with pie charts indicating the accumulation of each metabolite in the three organs (yellow shading for flowers, blue for pedicels, and green for immature fruits). The size of the node denotes the ion intensity detected in the negative ionization mode. Bar chart summarizes the percentage accumulation value of all nodes associated to the same “molecular family”.

The diversity and distribution of chemical classes (Figure S3) in the extracts of the three organs: flowers, pedicels, and immature fruits were investigated (Figure 3 and Figure 4). This analysis revealed that the highest structural diversity of compounds in the reproductive organs of Aesculus is mainly restricted to three “molecular families”, representing flavonoids, procyanidins and highly polymerized procyanidins (tannins) (Figure S4). Among the flavonoids, glycosylated derivatives of quercetin and kaempferol were preferentially accumulated in the flowers compared to the pedicels and immature fruits (Figure 3). This combined with the loading plot data (Figure 1b) confirms that kaempferol and quercetin derivatives are mainly a chemical character of flowers in the genus *Aesculus*. Further analysis of this molecular family indicated that a high structural diversity of glycosylated flavonoids is present in the flower extracts. Many of these compounds are still pending identification given that no spectral match was found in the GNPS database and in our in-house library of MS^2^ spectra, thus indicating that the flowers of species from *Aesculus* can be a potential source of new or unusual flavonoid glycosides. This analysis further conveys that the percentage accumulation of flavonoid glycosides in the flowers of *Aesculus* represents more than 50% of total accumulation of this chemical class in the three plant organs (Figure 3).

Analysis of the second and third more structurally diverse “molecular families,” representing epicatechin, procyanidins, and associated polymeric structures, revealed that they were predominantly found in fruit extracts compared to the pedicels and flowers (Figure 4). The percentage accumulation bar chart shows that on average 60% of flavan-3-ols and associated structures are accumulated in immature fruits, whereas the flowers and pedicels only had around 20% each. This combined with the loading plot data (Figure 3) confirms that procyanidins are a chemical character of fruit extracts.

### 2.2. Anti-Fungal Assays

**Figure 5:**
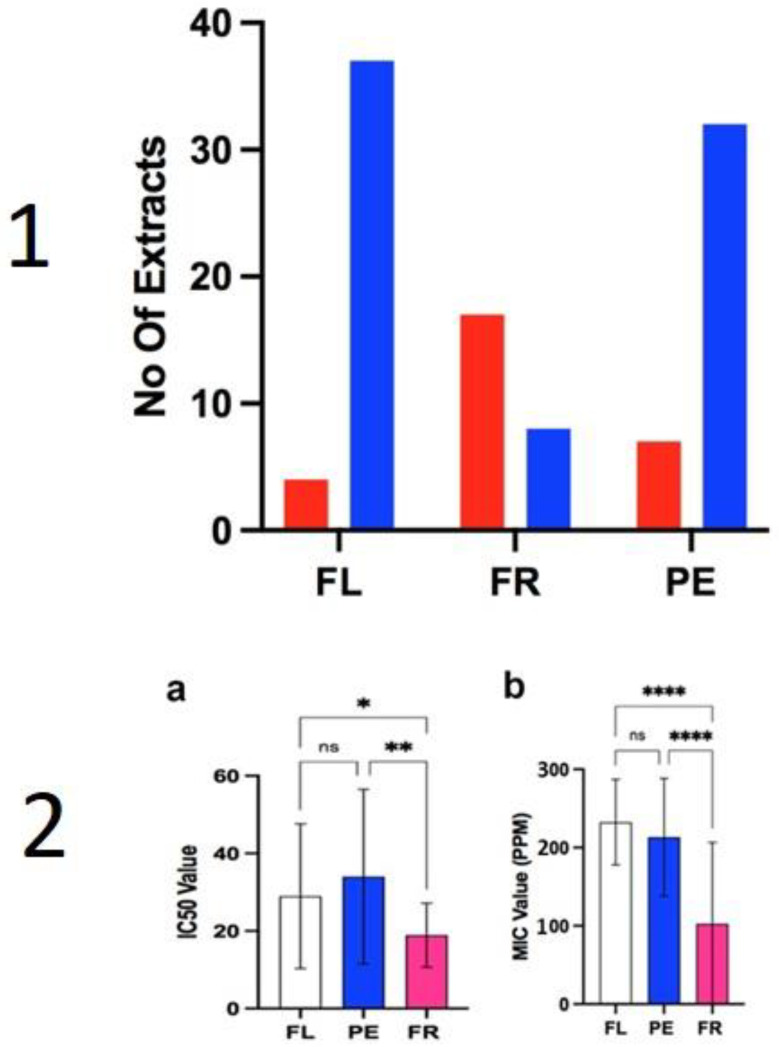
**Plate 1**: Number of active and inactive extracts against *Saccharomyces cerevisiae*. Extracts of *Aesculus* were tested against *Saccharomyces cerevisiae* in an MIC assay. Extracts that produced some form of inhibition at starting concentration of 250 µg·mL^−1^ were reported as active (red bars) whereas extracts that did not inhibit *S. cerevisiae* were recorded as inactive (blue bars). N = 111, FL = flowers, FR = fruits, PE = pedicels. **Plate 2**: mean IC_50_ values (**a**) and MIC values (**b**) for extracts of *Aesculus*, grouped according to organs. Antioxidant assays were performed in triplicate using the DPPH radical with the amount of color change measure spectrometrically using a plate reader at 517 nM. MIC assays were also performed in triplicate against *Saccharomyces cerevisiae* with the lowest concentration to produce inhibition recorded as the MIC value. FL (white) represent flowers, PE (blue) represents pedicels, FR (fushcia) represents fruits. Graphs are plotted as the mean +/− standard deviation. Some error bars are clipped at the axis limit. * denote the degrees of significance difference in a Kruskal–Wallis multiple comparison test; ns, not significant, * *p* < 0.5, ** *p* < 0.1 **** *p* < 0.0001.

The inhibitory activity of the extracts from *Aesculus* against the model yeast *Saccharomyces cerevisiae* (SAC) is provided as a minimum inhibitory concentration (MIC, µg·mL^−1^) averaged from three repeats (Table 2). A few extracts from flowers inhibited *S. cerevisiae*, but only at the starting concentration of 250 µg·mL^−1^, i.e., 10% of flower extracts showed inhibition, compared to 68% of extracts from immature fruits, and 18% of extracts from pedicels (Figure 5: plate 1). The averaged MIC values of extracts from the immature fruits were the most efficient (102 ± 20.8 µg·mL^−1^), followed by the pedicels (213 ± 75 µg·mL^−1^) and then the flowers (235 ± 54 µg·mL^−1^) (Figure 5: plate 2). This conveys that the strongest antifungal organ is the immature fruit.

Furthermore, the antifungal activity of the immature fruits was significantly higher according to a Kruskal–Wallis test, which showed a **** *p* < 0.0001 significant difference, compared to pedicels and flowers (Figure 5: plate 2). However, activity ranged significantly across different fruit extracts (Table 2 and Figure 5: plate 2) with a few extracts showing less inhibition than some flowers and pedicels, suggesting differences in their antifungal potential. Furthermore, a large standard deviation was determined from data of each of the organ’s extracts. In the case of the fruits a standard deviation of 208 µg·mL^−1^ was calculated, highlighting the range of activity across the fruit’s extracts.

### 2.3. Antioxidant Assays

To assay the radical scavenging ability of the extracts, a DPPH assay was performed. Each extract was tested in triplicate with the mean value taken. The inhibitory concentration IC_50_ value was calculated from a standard curve of known DPPH concentrations and reported in Table 2.

The data indicated that all the extracts had antioxidant effects but extracts from fruits showed a lower IC_50_ value at 18.93 ± 8.24 µg·mL^−1^, followed by the flowers 28.98 ± 18.65 µg·mL^−1^ and the pedicels 34.01 ± 22.50 µg·mL^−1^ (Figure 5: plate 2). Hence, extracts from fruits were significantly more active than flowers * *p* < 0.5 or pedicels ** *p* < 0.1 (Kruskal–Wallis test), with no significant difference between the flowers and pedicels (Figure 5: plate 2).

## 3. Discussion

### 3.1. Metabolomic Aspects of the Study

Our investigation set out to explore the chemical profiles of different species and organs within the genus *Aesculus* and their correlation with their antioxidant and antifungal activity. From chemical profiling across species and organs, it was possible to deduce that a strong chemical similarity is true across species, but not across organs from single specimens. Hence, chemical profiles of individual organs are a greater discriminator compared to species (Figure 1), i.e., metabolites were localized to certain organs. Kaempferol and quercetin derivatives are found mainly in the flowers, whereas epicatechin and procyanidins are predominantly in the fruits. In context, the clear chemical distinction between organs can be utilized to detect adulteration of mature fruits with pedicels, flowers, or immature fruits due to early harvesting, which could influence the quality of extracts produced, and may affect their industrial uses or clinical benefits. Furthermore, the high degree of similarities among species points to potential industry uses of other *Aesculus* that do not yet have any commercial implications. These chemical similarities could be due to a low genetic diversity across cultivated species of *Aesculus*, as previously demonstrated for other medicinally important crops [24]. However, further studies are necessary to prove this.

In comparison with published sources, the chemistry of reproductive organs has some overlap with the chemical profiles reported in other parts, such as the leaves [9,10]. Kaempferol and quercetin glycosides have all been previously reported in species of *Aesculus* and these earlier chemical analyses did not result in the discovery of new compounds. However, the presence of unknown compounds in the current study that eluted later in the chromatogram of pedicels may be worth considering in a future study. These four unknown compounds (Table 1) are key discriminators that indicate that pedicels are present in the extracted material, thus having important implications in the quality control of commercial products from *A. hippocastanum*. Clear chemical differences across different organs within the same plant have also been described in other medicinally important species [25].

Many of the compounds assigned in the current study have shown some clinical benefit in other studies, particularly quercetin, kaempferol, and procyanidins, which have demonstrated preliminary data as anti-cancer, anti-diabetic, and anti-inflammatory compounds [8,14,26,27,28]. Additionally, these compounds were studied in animal models and demonstrated tentative benefit for use as wound healing and neuroprotective agents [15,22]. However, it is important to consider that translation to human models may prove more changeling, due to bioavailability and kinetics of the compounds with the human body [29].

A variable that was not considered during the study was the potential impact of the health of trees, particularly the presence or absences of disease or pests such as *Cameraria ohridella*. Previous studies reported that this leaf miner affects the phenolic compounds in the leaves, which could account for some differences seen between the species [10]. However, the damaged verses undamaged leaves were not considered in the analysis of this study.

### 3.2. Antifungal Activity

The current study demonstrated that the immature fruits had greater ability to inhibit *S. cerevisiae* growth than the flowers or pedicels. In industry, mature fruits are used, so a possible point of further inquiry would be to examine the difference in bioactivity between mature fruits and immature fruits [7]. One limitation of the current study was that fruit collections were limited by comparison with flowers and pedicels, i.e., only 25 specimens were resampled when fruits developed. As anti-fungal activity was mainly confined to fruit extracts, the antifungal metabolites are possibly the flavan-3-ols, such as the procyanidins and catechins identified by LC–MS/MS and molecular networking; however, further isolation would be needed to confirm this. Studies also support the conclusion that catechins may be responsible for the antifungal effect, with similar compounds isolated from other species showing antifungal, microbial, and anti-viral effects [30]. Further evidence also points to the use of procyanidins as anticancer adjuvants by selectively inhibiting potassium channels to suppress tumor growth in rats [31].

The fruit extract of *A. hybrida* had an average MIC value of 20.9 µg·mL^−1^ against *S. cerevisiae*, which is comparable to that of the positive control nystatin, encouraging isolation work to identify the anti-fungal compound within the extract. Whilst published literature corroborates the anti-fungal effects in species of *Aesculus*, as observed in the current study, there are other broad-spectrum effects reported, which are against a range of bacteria, including Gram-negative organisms [32]. However, in the current investigation, preliminary screening with *Bacillus subtilis* and *Escherichia coli* did not reveal any activity at the highest concentration used (250 µg·mL^−1^), which may be associated with the type of extract that was produced in our study as mid-range polar extracts using EtOAc were produced, compared to other solvent extracts such as methanol [32].

### 3.3. Antioxidant Activity

The radical scavenging activity of the extracts followed a similar pattern as compared to the antifungal activity, with the fruit having the lowest mean IC50 value, followed by the flowers and lastly the pedicels. The correlation between antifungal and antioxidant effects is most likely a consequence of the B-type procyanidin content of the fruit, since this group of compounds is known for these effects [33].

Whilst the fruits were significantly different to the pedicels and flowers, no significant differences were seen between the flowers and pedicels. Furthermore, all the extracts exhibited some antioxidant activity, and a range of values was seen. The antioxidant effects seen in the study correlate with those of published work, although some differences have occurred due to the methodology and type of extract that was used in the study [26]. The high density of procyanidins may be an explanation for the higher activity in fruit extracts as this compound is often associated with antioxidant effects [21]. The large standard deviation that was seen not only in the antioxidant results, but the antifungal assay too, could also be explained by the minor difference in chemistry across species.

Overall, the results reiterate the uses of *Aesculus* as a plant of medical importance in both antifungal and antioxidant applications. Additionally, the presence of procyanidins, kaempferol, and quercetin derivatives suggest possible further uses as the reported literature points to anticancer antidiabetic, cardiovascular, and neuroprotective qualities of these isolated compounds [8,14,27,28]. The antioxidant ability of extracts shows that historical treatment for vascular disorders has some basis by reducing the oxidative strain [21]. Nevertheless, the work provides the basis for further work on the reproductive organs of *Aesculus*.

## 4. Materials and Methods

### 4.1. Samples

The organs from species of *Aesculus* were collected over late May to July 2019 from Kew Gardens, London UK (details in Table S1). Each species was then number coded (Table S1) and the flowers (FL), immature fruits (FR), and peduncles (PE) were separated and freeze dried to drive out the moisture. Extracts were then made from between 5–10 g of dried material with 75–80 mL of 80% methanol (MeOH) and 20% distilled water (all reagents were provided by Sigma–Aldrich, Saint Louis, MO, USA]. The extracted material was then re-extracted using pure ethyl acetate (EtOAc). The 80% MeOH extract was chemically the same as the EtOAc extract but was comprised of a significantly higher sugar content, so in going forward only the EtOAc extract was used. Following extraction, the solvents were removed using a rotary evaporator with further evaporation in a Genevac.

### 4.2. Liquid Chromatography–Tandem Mass Spectrometry (LC-MS/MS)

The EtOAc extracts were redissolved in MeOH to 10 mg per mL and filtered into 2 mL vials for liquid chromatography–mass spectrometry (LC–MS). LC–MS analyses were performed on an UltiMate 3000 Standard (SD) HPLC system (Thermo Scientific, Waltham, MA, USA) coupled to a 100 Hz diode array detector (DAD) and an Ion trap Velos Pro (Thermo Scientific, Waltham, MA, USA) mass spectrometer. Chromatographic separation of plant extracts (4 µL) was performed on a Luna C18 column (150 mm × 3 mm i.d., 3 μm, Phenomenex, Torrance, CA, USA) using a mobile phase gradient of 0:90:10 to 90:0:10 (MeOH: water: acetonitrile + 1% formic acid) over 20 min. 90% A was held for 5 min and then returned to initial conditions over 2 min, at 30°C (flow rate: 400 μL/min). UV detection was recorded between 190 and 450 nm.

Mass spectrometry detection was performed in both positive and negative ionization modes using the full scan and data-dependent MS^2^ and MS^3^ acquisition methods. Total ion current (TIC) chromatograms were obtained over the range of 125–2000 *m/z* using a spray voltage of +3.0 kV and −2.5 kV for the positive and negative ionization modes, respectively. Four different scan events were recorded for each ionization mode as follows: (1) Full scan; (2) MS^2^ of the most intense ion in scan event 1; (3) MS^3^ of the most intense ion in scan event 2, and (4) MS^3^ of the second most intense ion in scan event 2. Additional parameters for the mass spectrometer include capillary temperature, 300 °C; heater temperature, 300 °C; sheath gas flow rate, 60; auxiliary gas flow rate, 20; automatic gain control (AGC) target, 3.0 × 10^4^ (full scan) and 1.0 × 10^4^ (MS^n^); normalized collision energy for MS^n^, 35 eV; minimal signal required, 500 and isolation width, 4. Nitrogen was used as the drying, nebulizer and fragmentation gas.

### 4.3. Feature-Based Molecular Networking

Feature-based molecular networking (FBMN) was created following the workflow by Nothias et al., [34] on the GNPS platform (https://gnps.ucsd.edu, accessed on 4 September 2021). Chromatographic data in raw format of the negative ionization mode were transformed to mzXML format using the MSConvert package from the software ProteoWizard 3.0.9798 (Proteowizard Software Foundation, Palo Alto, CA, USA). The mass spectrometry data were then processed with MZmine 2.53 [35], and the results were exported to GNPS for FBMN analysis. Detailed parameters used in MZmine are described in the study of Padilla-González et al. [36].

For FBMN, the data were filtered by removing all MS/MS fragment ions within +/− 17 Da of the precursor *m/z*. MS/MS spectra were window-filtered by choosing only the top 6 fragment ions in the +/− 50 Da window throughout the spectrum. The precursor and fragment ion mass tolerance were both set to 2.0 Da. A molecular network was then created where edges were filtered to have a cosine score above 0.65 and more than 6 matched peaks. Furthermore, edges between two nodes were kept in the network only if each of the nodes appeared in each other’s respective top 10 most similar nodes. Finally, the maximum size of a molecular family was set to 100, and the lowest-scoring edges were removed from molecular families until the molecular family size was below this threshold. The spectra in the network were then searched against GNPS spectral libraries. The library spectra were filtered in the same manner as the input data. All matches kept between network spectra, and library spectra were required to have a score above 0.7 and at least 4 matched peaks. The molecular networks were visualized using the software Cytoscape [37].

To confirm and expand the spectral library annotation made by molecular networking, accurate mass values, MS/MS fragment ions, and UV spectra of the detected metabolites were manually inspected and compared with literature data and information available in our in-house library of MS^2^ spectra. To have an overview of the confidence level achieved in the identification of metabolites, we adopted the four levels of accuracy reported in the Metabolomics Standard Initiative [38].

### 4.4. Inhibition of Saccharomyces Cerevisiae

To assess the extracts’ anti-fungal ability a minimum inhibitory concentration (MIC) assay was performed followed the clinical laboratory standards protocol in a 96 well microtiter plate [39]. The organism *Saccharomyces cerevisiae* was chosen because of its widespread use in mechanistic studies. The availability of genetic mutants in our laboratory can be used in follow up studies for confirmation of the mechanism of inhibition.

The 10 µL of a methanol solution at 10 mg.mL^−1^ was combined with 10 µL of dimethyl sulfoxide (DMSO) and 200 µL of SC broth to give a final concentration of 500 µg·mL^−1^, that becomes 250 µg·mL^−1^ after inoculation. The extracts were then serially diluted across six wells. Once diluted the wells were inoculated with 100 µL of SC broth made up with *S. cerevisiae* colonies to an approximant ocular density of 0.5 λ at 517 nm wavelength. The plates were then incubated for 24 h at 30 degrees Celsius and observed for inhibition, with the lowest µg·mL^−1^ value to inhibit *S. cerevisiae* recorded. Nystatin was used as a positive control. Furthermore, a negative control of MeOH was used.

### 4.5. Antioxidant DPPH Radical Assay

The free radical scavenging activity of the extracts was determined using a DPPH free radical assay in a 96 well microtiter plate. The starting concentration of the extracts was at 125 µg·mL^−1^ before combining with the DPPH solution, which gave a final starting concentration of 62.5 µg·mL^−1^. The extract was serially diluted two-fold across six wells, using an 80:20 (dimethyl sulfoxide: distilled water) solution of aqueous DMSO. The DPPH radical was made to a final concentration of 10 nM per L in MeOH and combined at an equal volume with the diluted extracts in the 96 well plate, then allowed to react for 10 min. Quenching of the DPPH converted its colour from purple to yellow and the degree of colour change was measured using a plate reader at 517 nm. The obtained values were then compared to a calibration curve of known DPPH concentrations to find the concentration at which 50% of the radical had been quenched. This value was then reported as the inhibitory concentration value (IC_50_). With each plate a negative control with pure MeOH was included to show that the effects of DPPH were only as a result of the extracts and not background. A positive control with a pure rutin sample was also used to monitor the DPPH reactions and determine the plateau point.

Known DPPH concentrations were created using a serial dilution of a 10 nM per L in MeOH solution and performed in triplet to obtain a mean value used in the calibration curve. Background absorbance from the plate was measured for pure MeOH, and this was subtracted before the absorbance values were measured. From the calibration curve, the extract IC_50_ values were determined by removing the background absorption, diving by the gradient of the calibration line and finding the mid-point for the 50% in calibration curve, which was used to determine concentration for the extracts. The mean IC_50_ from three assays with the DPPH radical was then reported as an average.

### 4.6. Data Analysis

The LCMS data obtained over the course of the study were visualized using Thermo Scientific Xcalibur software version 1.0. Peaks were the chosen by eye and compare to a Kew database of compound for identification. Additionally, published literature was used to assign peaks in the samples with the relative sizes (small, medium, large) of each record. Principle component analysis (PCA) and molecular networking analysis was also performed on the data using R statistics software version 1.0. MIC, antioxidant, and the DPPH calibration curve values were transformed using Excel software. The MIC and IC50 values were further analyzed and plotted by Graph Pad prism software (Version 9.4, Los Angeles, CA, USA) using a Spiro–Wilk normality test and Kruskal–Wallis test. All data are reported as mean standard deviation unless specified. Ns, not significant, * *p* < 0.5 ** *p* < 0.01 *** *p* < 0.001 **** *p* < 0.0001.

## 5. Conclusions

In conclusion, species within the genus *Aesculus* demonstrate a high degree of chemical consistency. However, the chemistry is significantly different between the organs. Within the current study, the antioxidant and antifungal activity was strongest when extracts from fruits were used. The activity of the fruit’s extracts may be explained in part by the presence of procyanidins, which are known to have antifungal and antioxidant effects.

## Figures and Tables

**Table 1:** Discriminant metabolites of different plant organs in a selection of taxa from the genus *Aesculus*. Metabolites are selected based on the analysis of the OPLS-DA loadings plot. Metabolites sorted by their added contribution in the first two components. Negative ionization mode used.

Rt (min)	*m/z*	Organ	Tentative Identification	MS^n^ Ions	Conf Level *
11.13	609.39	flowers	rutin	609 → 343, 301 bp, 300, 271, 255, 179	2
12.39	593.35	flowers	kaempferol *O*-rutinoside isomer 2	593 → 285 bp → 267, 257 bp, 241, 229, 213	3
11.38	463.37	flowers	quercetin 3-*O*-hexoside	463 → 301 bp → 271, 255, 179 bp, 151	2
12.55	447.37	flowers	quercetin 3-*O*-rhamnoside	447 → 301 bp → 271, 255, 179 bp, 151	2
16.04	285.67	flowers	kaempferol	285 → 284, 257, 229, 213, 151	2
12.22	593.35	flowers	kaempferol *O*-rutinoside isomer 1	593 → 285 bp → 267, 257, 255 bp, 241, 229, 213	3
10.29	739.42	flowers	kaempferol *O*-rutinoside-*O*-rhamnoside	739 → 693, 593, 575 bp, 473, 393, 327, 285, 227, 255, 285 → 255 bp, 241, 227, 213, 151	3
7.93	289.45	fruits	epicatechin	289 → 271, 245 bp, 205, 179	2
6.82	577.36	fruits	procyanidin B	577 → 559, 451, 425 bp, 407, 299, 289, 245	3
7.94	579.32	fruits	dimer of epicatechin	579 → 289 bp → 271, 245 bp, 205, 179	2
7.62	865.39	fruits	procyanidin trimer	856 → 847, 739, 713, 695 bp, 587, 577, 575, 543, 451, 449, 425, 407, 287, 245	3
1.80	191.63	fruits	quinic acid	191 → 173, 155, 127, 111, 99, 93, 85 bp, 71, 59	2
7.30	863.39	fruits	aesculitannin or isomer	863 → 711 bp, 693, 573, 559, 541, 531, 451, 411, 299, 289, 285	3
1.80	383.31	fruits	dimer of quinic acid	383 → 191 bp → 173, 155, 127, 111, 99, 93, 85 bp, 71, 59	2
1.69	387.31	pedicels	sucrose [FA adduct]	341 → 179 bp, 161, 143, 131, 119, 113, 101	2
22.25	559.59	pedicels	n.i.	559 → 513 bp, 277, 253	4
1.69	341.97	pedicels	sucrose	341 → 179 bp, 161, 143, 131, 119, 113, 101	2
1.69	683.36	pedicels	dimer of sucrose	683 → 341 bp → 179 bp, 161, 143, 131, 119, 113, 101	2
17.07	779.46	pedicels	n.i. [FA adduct]	733 → 651, 633 bp, 375, 357	4
1.77	533.28	pedicels	quinic acid glucoside	533 → 191 bp → 173, 155, 127, 111, 99, 93, 85 bp, 71, 59	2
13.18	423.48	pedicels	n.i.	423 → 279 bp, 249, 205, 169, 139	4
26.57	819.70	pedicels	n.i.	819 → 773, 513, 277 bp	4

* Confidence level achieved in the identification of metabolites: 1 (high), identified by retention time (Rt) and accurate MS comparisons with a reference standard; 2 (intermediate), identified by accurate MS comparisons and database searches in our reference library of MS2 spectra (Kew Library), in the Dictionary of Natural Products and by interpretation of fragmentation patterns; 3 (low), identity or chemical class suggested by accurate MS comparisons with database searches; 4 (lowest), unknown metabolites. n.i.: not identified; FA: formic acid.

**Table 2:** Mean DPPH IC_50_ values (µg·mL^−1^ of extract) and MIC values (µg·mL^−1^) for range extracts of *Aesculus*.

				Flowers		Fruits		Pedicel	
Species	Authority	Replicate No.	Code	DPPH	MIC	DPPH	MIC	DPPH	MIC
*A. hippocastanum*	L.	1	11	20.5	n.i *	11.6	n.i	33.7	n.i
		2	12	41	n.i	14.8	n.i	20	n.i
		3	24	33.9	n.i	- *	-	138	n.i
		4	25	-	n.i	11.7	125	12.1	n.i
*A. hybrida*	DC.	1	10	38.5	n.i	19.9	n.i	42.2	n.i
		2	30	29	n.i	12	20.85	30	n.i
*A. flava*	Sol.	1	14	6	n.i	46.2	125	43.2	n.i
		2	4	9.8	n.i	19.6	62.5	40.5	n.i
*A. indica*	(Wall. ex Cambess.) Hook	1	33	29.8	208.35	-	-	14.3	n.i
		2	23	22.1	n.i	-	-	23.1	n.i
*A. chinensis*	(Rehder) Turland and N.H.Xia	1	28	22.6	n.i	-	-	5.5	n.i
		2	35	14.1	n.i	-	-	35.4	n.i
*A. assamica*	Griff	1	38	41.5	n.i	-	-	52.1	n.i
		2	31	35.7	n.i	15.2	62.5	16.7	n.i
*A. turbinata*	Blume	1	34	9.1	n.i	15.4	n.i	12.2	n.i
		2	6	52.3	n.i	13.6	n.i	23.5	n.i
*A. californica*	(Spach) Nutt.	1	36	45.2	166.65	-	-	32.2	n.i
		2	37	48.8	125	-	-	1289.7	n.i
*A. parviflora*	Walter	1	39	53.3	n.i	16.2	n.i	34.1	n.i
		2	40	47	n.i	-	-	43.3	n.i
*A. neglecta*	Lindl.	1	7	11.1	62.5	33.9	n.i	5.5	166.65
		2	18	-	-	19.4	83.35	33.2	n.i
*A. mutabilis*	(Spach) Scheele	1	8	15.4	n.i	-	-	22.2	166.65
		2	17	24.8	n.i	23.5	41.65	31.7	n.i
*A. carnea*	Zeyh **	1	21	42.3	n.i	29.3	31.25	22	n.i
		2	32	19.3	n.i	15.9	62.5	7.8	n.i
		3	9	28.2	n.i	15.2	n.i	43.3	208.35
		4	9	37.2	n.i	-	-	-	-
		5	29	46.1	n.i	9.4	83.35	37.4	n.i
*A. sylvatica*	W.Bartram	1	16/20	19.3	n.i	-	-	-	-
		2	20	19.7	n.i	-	-	28.4	83.35
		3	16	8.2	n.i	-	-	48.1	125
*A. glabra*	(Buckley) Rob.	1	29	26	n.i	-	-	-	-
		2	26	45.3	n.i	18.3	62.5	39.9	n.i
		3	19	18.1	n.i	23.2	31.25	48	n.i
		4	22	40.3	n.i	10.5	62.5	24.4	n.i
		5	2	4.1	n.i	-	-	24.6	n.i
		6	3	5.6	n.i	-	-	69.3	166.65
*A. dallimorei*	Sealy **	1	13	98.6	n.i	-	-	48	n.i
		2	27	16.2	n.i	16.7	125	51.8	n.i
*A. marylandica*	Booth ex Dippel	1	5	3.7	n.i	27.5	62.5	30.6	n.i
*A. bushii*	C.K.Schneid	1	15	45.8	n.i	19.2	41.65	-	83.35
*A. pavia*	L.	1	1	12.5	n.i	15	41.65	24	62.5

* n.i = No inhibition, which indicates that the extract was not tested or a value has not been possible to calculate. ** indicates that there is taxonomic uncertainty about this determination according to the world checklist of vascular plants. MIC represent the mean minimum inhibitory concentration to inhibit growth of *Saccharomyces cerevisiae* in µg·mL^−1^. DPPH represent the mean inhibitory value at which 50% of DPPH radicals are quenched (IC_50_) in µg·mL^−1^ of extract.

## Data Availability

Not applicable.

## Supplementary Materials

The following are available online at https://www.mdpi.com/article/10.3390/plants10122695/s1, Figure S1: Mass spectra of quality control *Aesculus* sample (Negative mode). A quality control sample that comprised of 10 µL of each extract of *Aesculus* was run in the LC-MS to monitor quality and assist in the identification of chemical peaks. Numbers 1–14 represent identifiable peaks within the sample (absolute mass), 1 = Quinic acid (192), 2 = Protocatechuic acid hexoside (316), 3 = 3-*O*-caffeoylquinic acid (354), 4 = Procyanidin B1 (578), 5 = (+)-catechin (290), 6 = Di-Galloyl-Arbutin (576), 7 = Rutin (610), 8 = Quercetin-3-*O*-glucoside (463), 9 = Quercetin-3-*O*-arabinoside (433), 10 = Kaempferol-3-*O*-rutinoside (593), 11 = Keampferol-3-*O*-arabinoside (417) 12 = Keampferol-3-*O*-rhamnoside (431), 13 = Isorhamnetin-3-*O*-glucoside (477), 14 = Isorhamnetin-3-*O*-pentoside (477), Figure S2: LC–MS spectra of a selection of species of Aesculus Flowers. The flowers of five species of *Aesculus* (tree identity numbers 1–5) were extracted in ethyl acetate and analyzed by LC-MS. The selected spectra show a high degree of similarity across the species of *Aesculus* which was mirrored in other samples and organs across species, Figure S3: Global molecular network gross, Figure S4: Chemical structures of some of the major compounds identified by mass spectrometric analysis, Table S1: Species, authority and the LivCol numbers associated with collections made in the Royal Botanic Gardens, Kew, Richmond, UK.
