# Supplementary material for: Comparative Metabolomics of Reproductive Organs in the Genus Aesculus (Sapindaceae) Reveals That Immature Fruits Are a Key Organ of Procyanidin Accumulation and Bioactivity
Source: Plants (Basel). 2021 Dec 8;10(12):2695. doi: 10.3390/plants10122695 (PMC8708636; doi:10.3390/plants10122695)
Supplement: Supplementary file 1 [file plants-10-02695-s001.zip › plants-1463650-supplementary.pdf]

Supplementary Files: **Comparative metabolomics of reproductive organs in the genus *Aesculus* (Sapindaceae) reveals that fruits are a key organ of procyanidin accumulation and bioactivity**

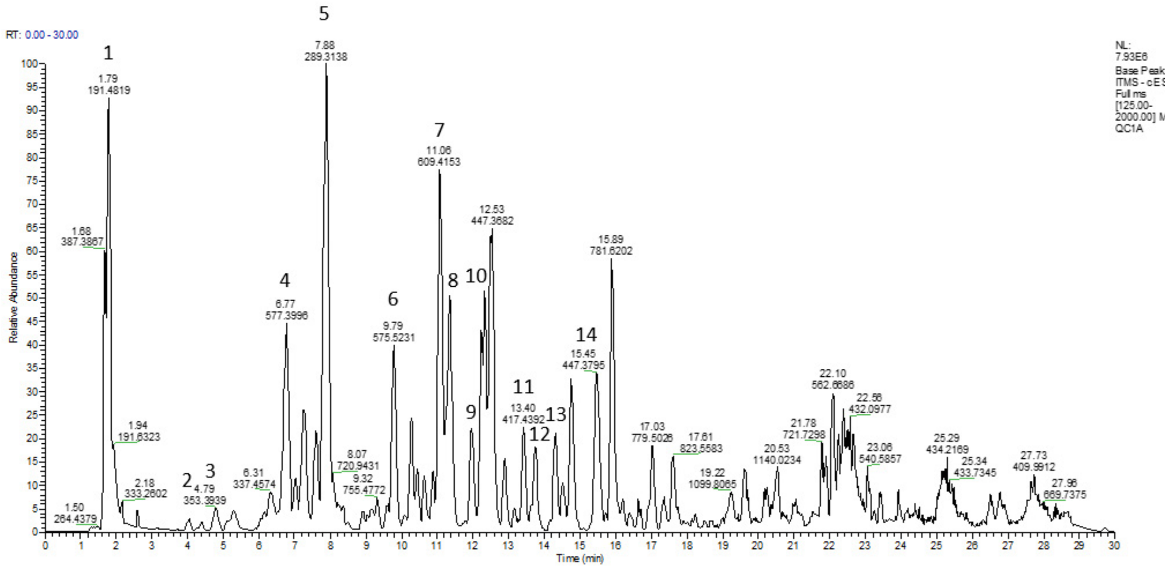

Figure S1. **Mass Spectra of quality control *Aesculus* sample (Negative mode).** A quality control sample that comprised of 10 μL of each extract of *Aesculus* was run in the LC-MS to monitor quality and assist in the identification of chemical peaks. Numbers 1-14 represent identifiable peaks within the sample (absolute mass), 1= Quinic acid (192), 2= Protocatechuic acid hexoside (316), 3= 3-*O*-caffeoylquinic acid (354), 4= Procyanidin B1 (578), 5= (+)-catechin (290), 6=Di-Galloyl-Arbutin (576), 7=Rutin (610), 8=Quercetin-3-*O*-glucoside (463), 9=Quercetin-3-*O*-arabinoside (433), 10=Kaempferol-3-*O*-rutinoside (593), 11=Keampferol-3-*O*-arabinoside (417) 12=Keampferol-3-*O*-rhamnoside (431), 13=Isorhamnetin-3-*O*-glucoside (477), 14=Isorhamnetin-3-*O*-pentoside (477).

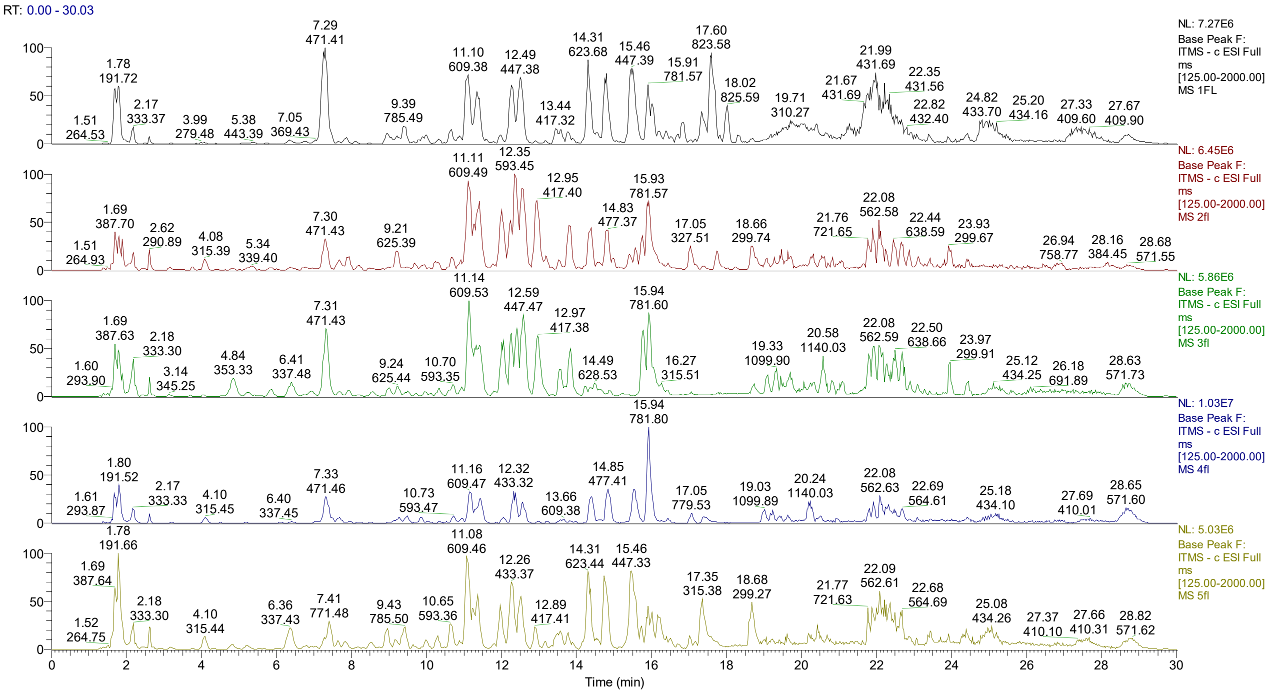

Figure S2. LC-MS Spectra of a selection of species of *Aesculus* Flowers. The flowers of five species of *Aesculus* (tree identity numbers 1-5) were extracted in ethyl acetate and analysed by LC-MS. The selected spectra show a high degree of similarity across the species of *Aesculus* which was mirrored in other samples and organs across species.

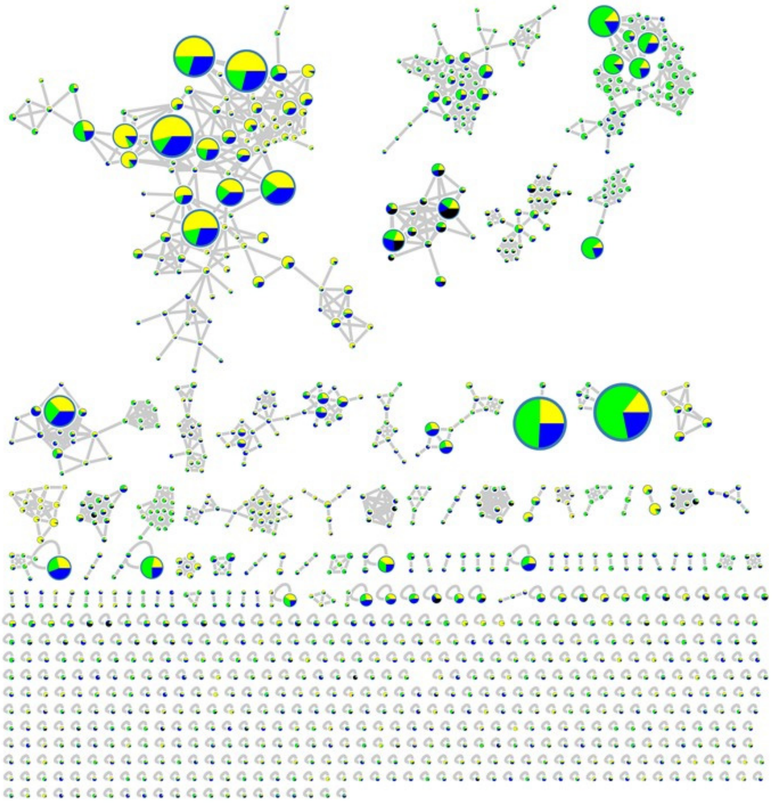

Figure S3. Global molecular network gross.

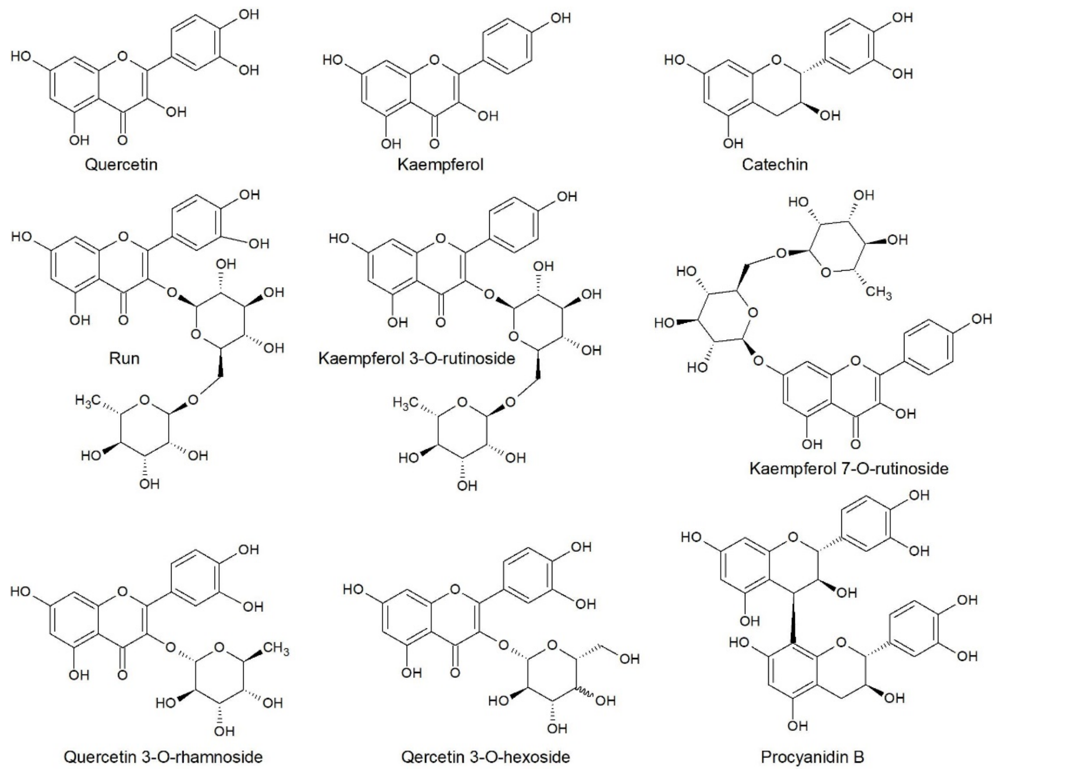

Figure S4. Chemical structures of some of the major compounds identified by mass spectrometric analysis.

Table S1. Species, authority and the LivCol numbers associated with collections made in the Royal Botanic Gardens, Kew, Richmond, UK.

Genus	Species	Authority	Tree Identification***	Identification Number
Aesculus	*pavia* 'Humilis'	L.	1969-10445	1
Aesculus	*glabra* 'Arguta'	(Buckley) Rob.	1987-8245	2
Aesculus	*glabra* 'Arguta'	(Buckley) Rob.	1969-10414	3
Aesculus	*flava*	Sol.	1912-23909	4
Aesculus	*marylandica*	Booth ex Dippel	1969-10428	5
Aesculus	*turbinata*	Blume	1887-58002	6
Aesculus	*neglecta*	Lindl.	1924-72102	7
Aesculus	*mutabilis*	(Spach) Scheele	1969-10431	8
Aesculus	*carnea* 'plantierensis'	Andre*	1900-1211	9
Aesculus	*hybrida*	DC.	1969-10427	10
Aesculus	*hippocastanum* 'memmingeri'	K.Koch	1884-17501	11
Aesculus	*hippocastanum*	L.	1969-17282	12
Aesculus	*dallimorei*	Sealy**	2010-1835	13
Aesculus	*flava*	Sol.	1871-40102	14
Aesculus	*bushii*	C.K.Schneid	1921-32801	15
Aesculus	*sylvatica*	W.Bartram	1924-72116	16
Aesculus	*mutabilis*	(Spach) Scheele	1983-5	17
Aesculus	*neglecta*	Lindl.	1924-72102	18
Aesculus	*glabra* 'OH 10 Buckeye'	Willd.	1922-45402	19
Aesculus	*sylvatica*	W.Bartram	1969-10433	20
Aesculus	*carnea*	Zeyh*	1969-12611	21
Aesculus	*glabra* 'glabra'	Willd.	2014-167	22
Aesculus	*indica* "Sydney Pearce"	(Wall, ex Cambess.) Hook.	1973-14434	23
Aesculus	*hippocastanum* 'beaumanii'	(C.K.Scheid.) Dole	1876-7601	24

Aesculus	*hippocastanum* 'pyramidailis'	(C.K.Scheid.) Dole	1896-57102	25
Aesculus	*glabra* 'glabra'	Willd.	1912-66401	26
Aesculus	*dallimorei*	Sealy**	1963-52601	27
Aesculus	*chinensis 'willsonii'*	(Rehder) Turland and N.H.Xia	1916-7601	28
Aesculus	*carnea*	Zeyh*	1969-10403	29
Aesculus	*hybrida*	DC.	1969-10440	30
Aesculus	*assamica*	Griff	1924-1607	31
Aesculus	x *carnea*	Zeyh*	1969-10402	32
Aesculus	*indica*	(Wall. ex Cambess.) Hook	1986-2811	33
Aesculus	*turbinata*	Blume	1969-10450	34
Aesculus	*chinensis*	Bunge	1913-52819	35
Aesculus	*californica*	(Spach) Nutt.	2001-4452	36
Aesculus	*californica*	(Spach) Nutt.	1982-145	37
Aesculus	*assamica*	Griff	1924-1601	38
Aesculus	*parviflora*	Walter	1969-10442	39
Aesculus	*parviflora*	Walter	1998-3139	40

* *A carnea* 'plantierensis' is synonym of Aesculus x carnea Zehy ** This is an unplaced name *** Tree Identification Number refers to the year the plant was planted and a locator number assigned by Royal Botanic Gardens, Kew.
